# Pre-emptive treatment in later-onset urea cycle disorders: a clinical perspective on glycerol phenylbutyrate

**DOI:** 10.1186/s13023-026-04466-4

**Published:** 2026-06-25

**Authors:** Marco Spada, Francesco Porta

**Affiliations:** 1 Department of Pediatrics, AOU Città della Salute e della Scienza di Torino, University of Torino, Torino, Italy; 2 Department of Clinical and Biological Sciences, University of Torino, Torino, Italy

**Keywords:** Urea cycle disorders, Glycerol phenylbutyrate, Later-onset urea cycle disorders

## Abstract

**Background:**

Later-onset urea cycle disorders (UCD) are characterized by variable clinical presentation and unpredictable metabolic decompensation. Current management often relies on a reactive approach, with treatment initiated after clinical manifestations. Glycerol phenylbutyrate (GPB) is widely used for ammonia control in UCD. However, its role in later-onset forms remains incompletely defined, particularly regarding timing of initiation. Available evidence suggests that hyperammonemia-related neurological injury may occur even in apparently stable patients, raising concerns about the adequacy of a wait-and-see strategy. This work provides a clinical perspective integrating current evidence together with personal experience to explore the potential role of pre-emptive GPB therapy in later-onset UCD. The limitations of current approaches are discussed, highlighting the discrepancy between apparent biochemical stability and ongoing neurological risk.

**Conclusions:**

We suggest that a proactive treatment strategy may reduce the risk of neurological complications in selected patients with later-onset UCD. We propose a shift from reactive to pre-emptive management, positioning GPB as a potential tool in this context.

## Introduction

**Fig. 1:**
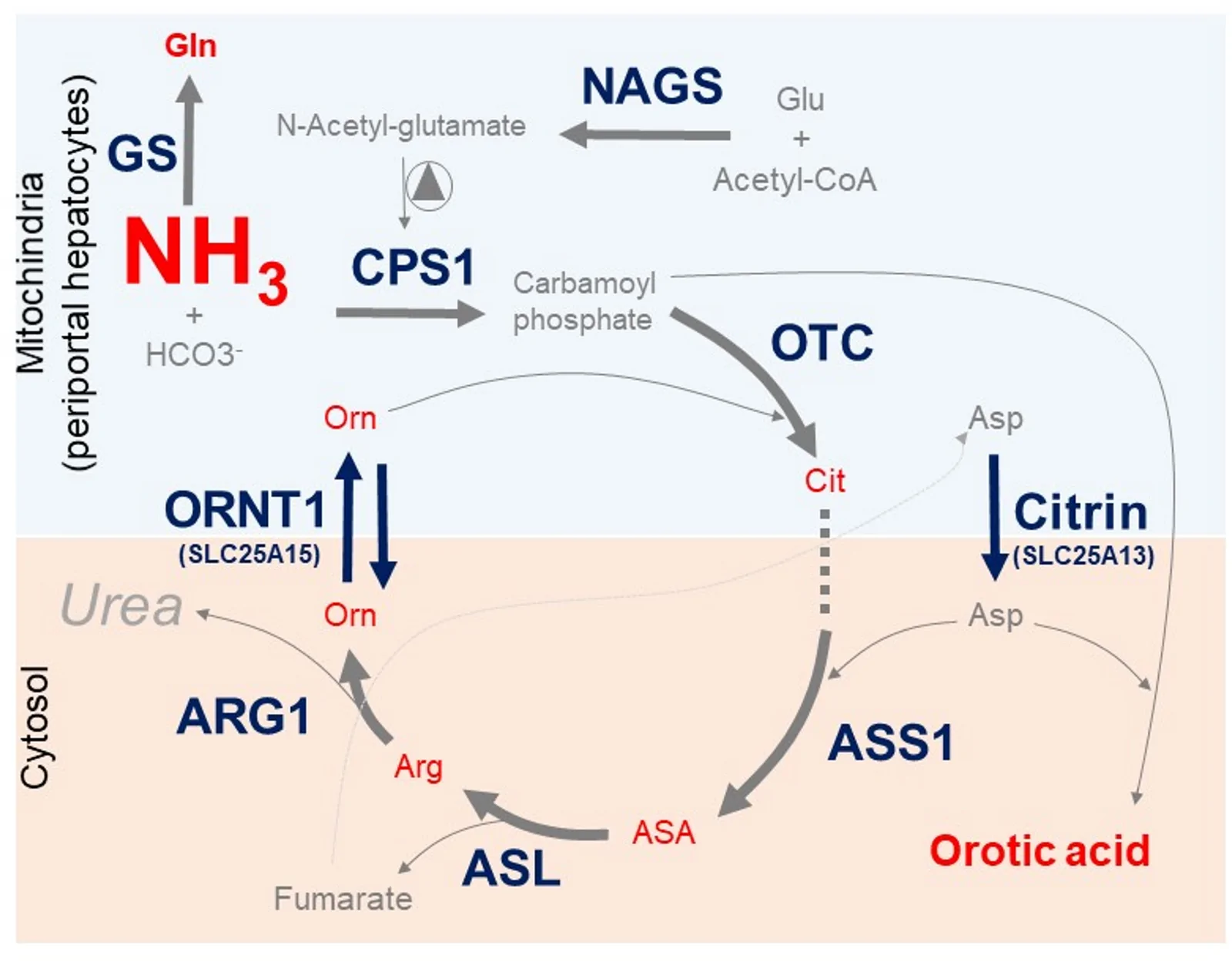
Schematic representation of the urea cycle. GS, glutamine synthetase; NH3, ammonia; HCO3-, bicarbonate; CPS1, Carbamoyl phosphate synthetase 1; NAGS, N-acetylglutamate synthase; Glu, glutamate; OTC, ornithine transcarbamylase; ASS1, arginosuccinate synthetase; ASA, arginosuccinic acid; ASL, arginosuccinate lyase; ARG1, arginase 1

**Fig. 2:**
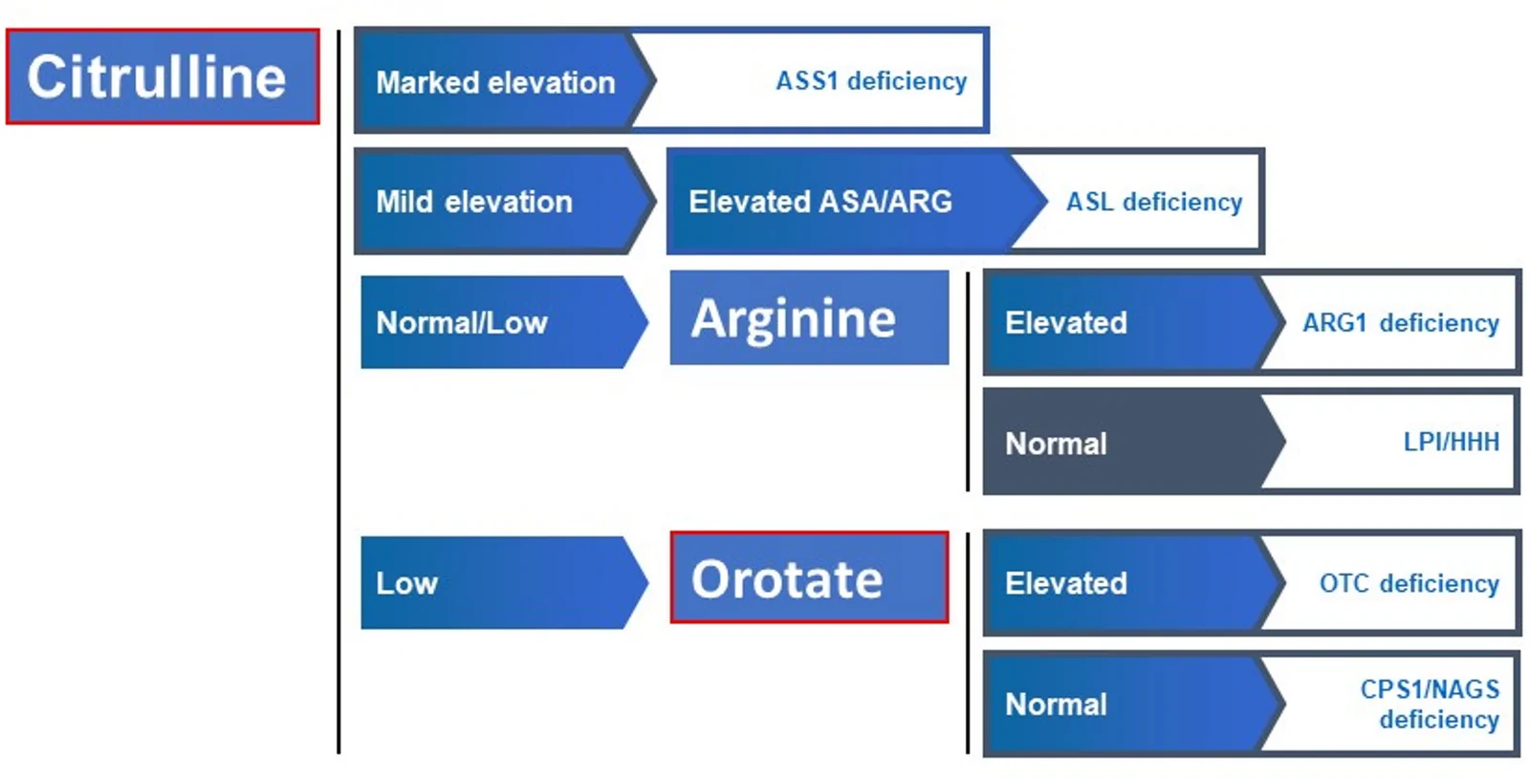
Differential diagnosis of urea cycle disorders (UCD) according to their biochemical profile. ASS1, arginosuccinate synthetase; ASL, arginosuccinate lyase; ARG1, arginase 1; LPI: lusinuric protein intolerance; HHH: Hy-perornithinemia–Hyperammonemia-Homocitrullinuria syndrome; OTC, ornithine transcarbamylase; CPS1, Carbamoyl phosphate synthetase 1; NAGS, N- acetylglutamate synthase

Urea cycle disorders (UCD) are inherited metabolic conditions hindering the detoxification of ammonia, with an overall incidence of 1 in 35,000 live births [1]. With the exception of ornithine transcarbamylase (OTC) deficiency, an X-linked recessive condition, UCD are autosomal recessive disorders. Transatlantic and European studies identified large cohorts of patients receiving professional care for UCD, with over 200 cases documented in Italy [2–4]. Clinical presentation of UCD varies widely, ranging from severe life-threatening neonatal onset with hyperammonemic coma to later-onset variants that may remain asymptomatic until adulthood. Such variability largely reflects the involved metabolic step and the severity of the underlying enzymatic defect (Fig. 1) [5–8]. Ammonia neurotoxicity is partly mediated by glutamine accumulation, contributing to neuronal dysfunction and cerebral edema [9–12]. Both blood ammonia and glutamine levels are useful biochemical markers for the diagnosis and follow-up of patients with UCD, who present peculiar biochemical hallmarks (Fig. 2). In general, onset anticipation and severity of neonatal hyperammonemia is maximal in proximal UCD with complete metabolic deficiency [13]. This gradient is exemplified by ARG1 deficiency (i.e. the last step of urea cycle), in which hyperammonemia can be absent even in cases with complete enzyme deficiency [14]. Mild UCD variants with residual metabolic activity, on the other hand, can manifest later in life with acute or chronic symptoms mostly related to intermittent or remittent hyperammonemia. In these patients, acute life-threatening hyperammonemia can be triggered either by catabolic events (such as febrile illnesses, fasting, stress, or delivery) or protein overload [15]. The same applies to female carriers of OTC deficiency [16, 17]. Diagnosis of later-onset UCD can be non-obvious, as chronic clinical symptoms are subtle, including food aversion, neurocognitive difficulties, behavioral abnormalities, and gastrointestinal complaints [18]. Moreover, life-threatening acute hyperammonemia in later-onset UCD can occur without any previous sign of the disease. Expanded newborn screening may facilitate earlier recognition of selected UCD [19]. Timely diagnosis and optimized clinical management are essential to prevent clinical sequelae and improve long-term outcomes [20, 21]. Here, we discuss the potential role of pre-emptive glycerol phenylbutyrate (GPB) treatment in later-onset UCD, integrating current evidence with clinical experience.

## Materials and methods

A narrative review of the literature was conducted using PubMed as the primary database. Relevant articles were identified using combinations of the following search terms: “urea cycle disorders”, “UCD treatment”, “UCD diagnosis”, “glycerol phenylbutyrate”, and “UCD scavengers”. Pooled analyses, clinical trials, open-label studies, surveys, observational studies, and case reports published in English within the last 10 years were considered. Articles reporting clinical outcomes and real-world use of GPB in patients with UCD were preferentially included. Pre-clinical studies were excluded.

## Results

**Fig. 3:**
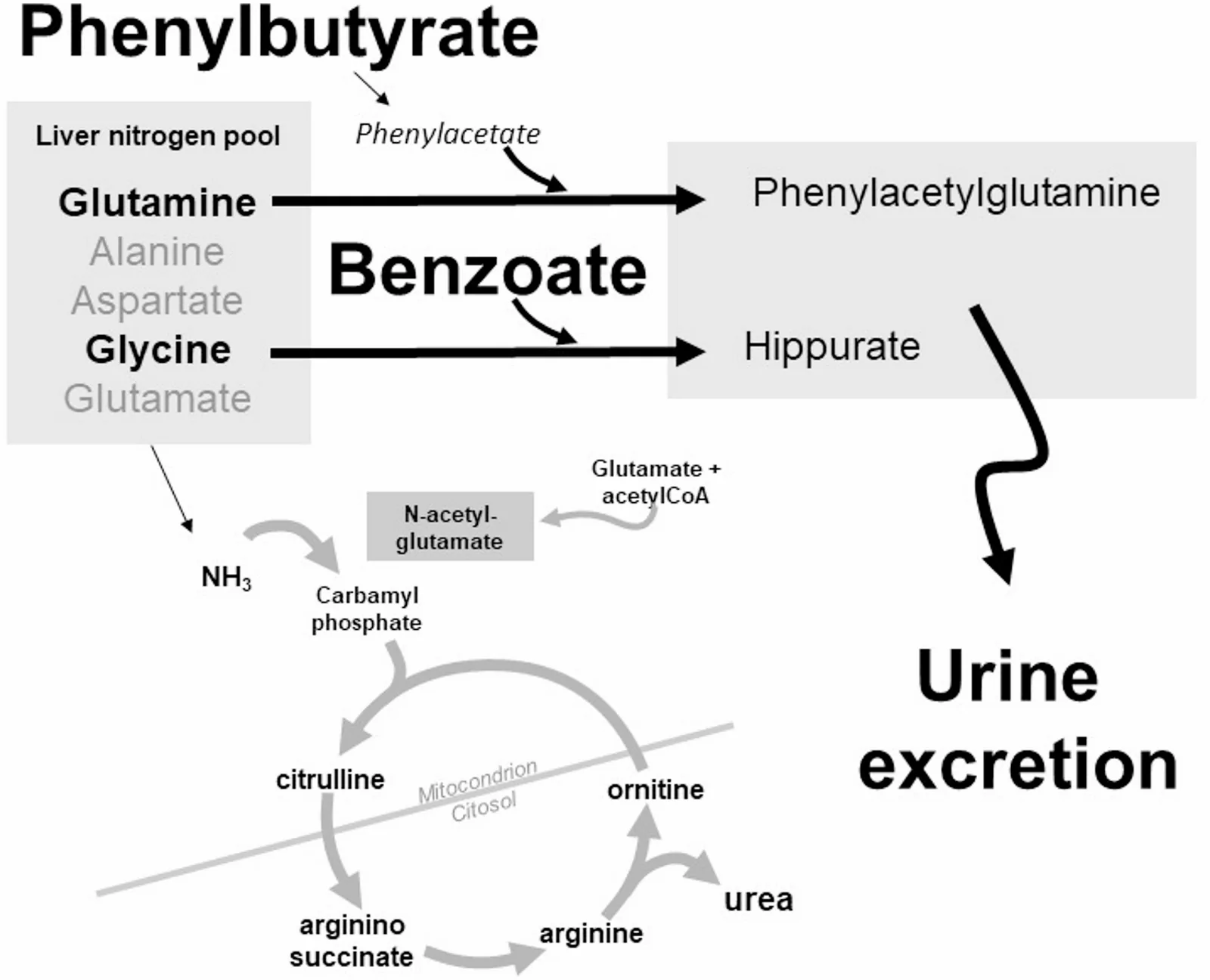
Mechanism of action of the nitrogen scavengers (sodium-) benzoate and phenylbutyrate (PBA). Glycerol phenylbutyrate (GPB) is hydrolyzed to PBA to exert its action

Current management of later-onset UCD remains heterogeneous, particularly in asymptomatic or mildly symptomatic patients. Current guidelines recommend maintaining plasma ammonia and glutamine within target ranges [18]. Liver transplantation is increasingly considered in severe neonatal-onset UCD, including carbamoyl phosphate synthetase I (CPS1), ornithine transcarbamylase (OTC), and argininosuccinate synthetase (ASS) deficiency. Recent studies have also shown benefits in severe argininosuccinate lyase (ASL) deficiency [22]. On the other hand, patients with later-onset UCD usually rely on long-term medical management [23]. Long-term management strategies remain highly variable, particularly in pre-symptomatic patients. Medical management commonly includes dietary protein restriction, specific amino acids supplementation, and nitrogen scavengers. Such clinical management, however, is frequently hampered by compliance issues with increased risk of recurrent life-threatening metabolic decompensations [24, 25]. Dietary management in UCD is highly variable across Europe, owing both to phenotypic variability and to the difficulty in balancing protein under- and over-restriction [26–28]. Amino acids supplementation may be beneficial in selected UCD depending on the specific enzymatic defect (Figs. 1 and 2) [29, 30]. Nitrogen scavengers remain a cornerstone of long-term UCD management. Oral formulations of sodium benzoate (NaBz) are currently not commercially available, so that the drug is administered orally off-label and hampered by poor compliance. On the other hand, oral formulations of sodium phenylbutyrate (NaPBA) are approved both in the US and in Europe [31, 32]. The comparative mechanisms of action of NaBz and NaPBA are shown in Fig. 3. These traditional nitrogen scavengers are effective but frequently limited by poor palatability and adherence [33]. In 2013, GPB was introduced to address the shortcomings of other scavengers and enhance nitrogen removal [34]. GPB is a tasteless and odorless oral liquid formulation designed to improve tolerability and long-term adherence. Comparative studies showed tighter ammonia control with GPB than with traditional scavengers [35]. By clearing three times more nitrogen than NaPBA, smaller volumes of GPB are required for effective treatment. Multiple studies evaluated the clinical effectiveness of GPB in patients with UCD (Table 1). Overall, GPB treatment has been associated with improved metabolic control, reduced risk of metabolic decompensation, and improved neurocognitive outcomes in longitudinal studies. Transitioning from other scavengers to GPB has also been associated with improvements in different health-related outcomes, including quality of life [41, 42, 53]. GPB allows individualized treatment strategies, with flexible dosing schedules and a broad therapeutic window, potentially improving long-term adherence and metabolic stability.

**Table 1:** Key features of recent studies on glycerol phenylbutyrate (GPB) treatment in patients with urea cycle disorders (UCD)

Reference	Type of study	Age, gender	Diagnosis	Naïve or transitioned to GPB from other scavengers	Other treatments	Outcomes	Adverse events
Berry et al., 2014 [46]	Pooled analysis of data from clinical trials	< 2->18 y, 34 females 15 males	28 OTC 9 ASS1 11 ASL 2 ARG1	NaPBA	Diet	Improved ammonia and glutamine levels	Vomiting, diarrhea, decreased appetite
Diaz et al., 2013 [48]	Phase 3 clinical trial (GPB vs. NaPBA)	6->18 y, 78 females 32 males	2 CPS1 97 OTC 3 ASS1 7 non-OTC	NaPBA	Diet	Improved ammonia and glutamine levels, improved executive function in long-term	Mild symptoms
Longo et al., 2021 [41]	Open-label study	0–2 m, 7 females 9 males	8 OTC 7 ASS1 1 ASL	10 NaPBA 7 NaBz/NaPA	Diet, amino acids	Controlled ammonia and glutamine levels	Diaper dermatitis, diarrhea, upper respiratory tract infection, gastroesophageal reflux disease
Diaz et al., 2019 [42]	Open-label study	1–61 y, NA	61 OTC 9 ASS1 13 ASL 2 ARG1 3 HHH	6 Naïve 82 NaPBA	Diet	Normal ammonia and glutamine levels	No increase of adverse events compared to the 12-months GPB treatment period
Berry et al., 2017 and 2018 [43, 44]	Open-label studies	2 m-2 y, 10 females 7 males	2 CPS1 3 OTC 5 ASS1 5 ASL 2 ARG1	1 NaBz 14 NaPBA	Diet, amino acids	Controlled ammonia and glutamine levels, reduced incidence of metabolic decompensation	Neutropenia, vomiting, diarrhea, pyrexia, hypophagia, cough, nasal congestion, rhinorrhea, rash
Smith et al., 2013 [47]	Open-label study	< 2->6 y, 7 females 8 males	3 OTC 3 ASS1 8 ASL 1 ARG1	NaPBA	Diet	Reduced ammonia levels	NA
Lichter-Konecki et al., 2011 [49]	Open-label study	6–17 y, 10 females 1 male	9 OTC 1 ASS1 1 ASL	NaPBA	Diet	Lower ammonia levels in GPB vs. NaPBA treatment	NA
Lee et al., 2010 [50]	Open-label study	> 18 y, 6 females 4 males	8 OTC 1 ASS1 1 HHH	NaPBA	Diet	Lower ammonia levels and PBA systemic	NA
Yeowell et al., 2021 [40]	Survey	3->11 y, 2 females 7 males	NA	4 NaBz 5 NaPBA	NA	Improved health and social participation	NA
Nagamani et al., 2015 [33]	Survey	< 2->18 y, 67 females 33 males	1 CPS1 69 OTC 12 ASS1 13 ASL 2 ARG1 3 HHH	NaPBA	NA	NA	Abdominal pain, nausea, burning
Yeo et al., 2023 [37]	Retrospective observational study	0–17 y 5 males 15 females	3 OTC 8 ASS1 6 ASL	11 NaBz 5 NaPBA 3 NaPBA+NaBz	Carglumic acid	Lower plasma ammonia and glutamine, Fewer HACs and hospitalizations, reduced sodium and PG exposure	Pancytopenia, fatigue/appetite loss
Martín-Hernández et al., 2022 [39]	Retrospective observational study	0->18 y, 29 females 19 males	3 CPS1 27 OTC 8 ASS1 9 ASL 1 ARG1	2 NaBz 46 NaPB	NA	Significant reduction of ammonia and glutamine levels and hospitalization rate	Hair thinning (1), poor weight gain (1)
Cen et al., 2026 [52]	Retrospective observational study	43 females 58 males	13 CPS1 35 OTC 25 ASS1 10 ALS 12 ARG1 6 HHH	13 Naive	Diet Citrulline, arginine	Stable blood ammonia and biochemical results	NA
Hejazi et al. 2026 [51]	Retrospective observational study	0–22 y, 20 females 17 males	3 OTC 11 ASS1 20 ALS 3 ARG1	8 NaBz 9 NaPB 9 NaPBA+NaBz	Diet, arginine	Reduction of amminia levels and hyperammonemic crises	None
Martín-Rivada et al., 2024 [35]	Case report	Newborn, male	OTC	Naive	Diet	Adequate growth and neurological development after 3.5-year follow-up Appropriate biochemical control	One episode of gastroenteritis with mild HA
Sacchini et al., 2023 [36]	Case reports	0-13.5 y; 3 females 3 males	4 OTC 1 ASS1 1 ASA	1 Naïve 1 NaBz 4 NaPB	Citrulline, arginine	Relevant metabolic improvement, resulting in better therapeutic compliance, fewer hospitalizations, and improved quality of life	Metabolic decompensations during infections
Baker et al., 2022 [16]	Case report	10 m, female	OTC	NaPBA	Diet	Good metabolic control	NA
Feigenbaum et al., 2022 [31]	Case report	27 y, female	OTC	NaBz	Diet	Good metabolic control (during pregnancy)	One metabolic decompensation after delivery
Abbott et al., 2022 [38]	Case report	64 y, female	OTC	Naïve	Diet, citrulline	Good metabolic control	One metabolic decompensation
Laemmle et al., 2016 [45]	Case report	16 y, male	OTC	NaBz and NaPBA	Diet, amino acids	Improved metabolic stability	One metabolic decompensation

## Discussion

Current management of later-onset UCD remains largely reactive despite the risk of preventable neurological injury. This clinical perspective proposes a re-evaluation of the current management strategies in later-onset UCD, integrating current evidence and clinical experience. Prompt diagnosis and adequate treatment are essential to optimize survival and long-term neurological outcomes in selected later-onset UCD. The Italian expanded newborn screening program does not reliably detect all UCD, as different disorders are not included in the panel. In particular, OTC deficiency, the most common UCD, is not a current target of the Italian panel due to the low specificity of neonatal hypocitrullinemia. These considerations highlight the importance of clinical reasoning in patients presenting with symptoms potentially associated with later-onset UCD. Besides proper biochemical testing, the assessment of the family history is essential to address the suspect of later-onset OTC deficiency both in hemizygous males and heterozygous females [54]. On the other hand, the inclusion of ASS1, ASL, and ARG1 deficiency in the Italian newborn screening program allows their potential early suspicion, while also raising challenges regarding management of mild asymptomatic patients. Clinical management of these patients remains heterogeneous, due to limited published evidence. This represents a limitation of this narrative review, which integrates available evidence on current clinical management of UCD patients with personal views based on clinical experience. In particular, literature search could be biased by the heterogeneous nature of the described cohorts, including patients with different enzymatic defects, metabolic severity, diagnostic settings, and treatments. We believe that regular clinical and biochemical monitoring should be routinely performed in patients with UCD, even if no treatment is administered. Dietary restriction, amino acids supplementation, and/or scavengers can be implemented based on clinical, biochemical, and molecular data. Long-term nutritional profile of UCD patients can be characterized by selective reduction of plasma branched chain amino acids, a condition potentially heralding metabolic decompensation [55, 56]. With this regard, current evidences suggest that GPB treatment may offer important clinical advantages, given its flexibility, tolerability, safety, and effectiveness. In particular, GPB therapy has been reported to be associated not only to improved metabolic control, but also to improved nutritional status in UCD patients [57, 58]. We suggest that these findings support consideration of pre-emptive GPB treatment in selected asymptomatic patients with later-onset UCD. In particular, we advise considering pre-emptive treatment especially in females with a familiar history of neonatal-onset OTC in male patients. On the other hand, current evidence suggest that treatment is unnecessary in mild citrullinemia [58]. To better define need of pre-emptive management in pre-symptomatic later-onset UCD patients, moreover, clinical risk score calculators may represent useful tools for clinicians [59]. Pre-emptive management in these patients requires adequate clinical supervision and follow-up in order to optimize clinical outcomes. In our view, clinical assessments could be complemented by periodical glutamine monitoring on dried blood spot, a minimally invasive tool supporting clinical follow-up and avoiding overtreatment of later-onset UCD patients. We suggest that this pre-emptive approach could overcome the traditional “wait-and-see” strategy in which GPB treatment is delayed after the first potentially life-threatening metabolic decompensation. We suggest that such management may also be advantageous during transition from pediatric to adult care. Ideally, the transition of patients with later-onset UCD involves physicians with expertise in the field of inborn errors of metabolism in an adult hospital setting. Since clinical transition is not uniformly available, adult patients with later-onset UCD frequently receive suboptimal follow-up with related clinical risks [60]. Delaying treatment until clinical manifestations may expose these patients to preventable neurological injury. We suggest that a pre-emptive GPB strategy may improve long-term clinical stability and reduce preventable neurological injury in selected patients with later-onset UCD, while improving long-term adherence and continuity of care. In this context, tailoring the transition from pediatric to adult care appears essential not only for optimizing clinical and metabolic outcomes, but also for meeting the expectations of adolescents and adults with inborn errors of metabolism.

## Conclusions

Current evidence suggests that patients with UCD may benefit from GPB treatment. Based on available evidence and clinical experience, we suggest that a pre-emptive GPB strategy may reduce the risk of metabolic instability and prevent avoidable neurological injury in patients with later-onset UCD. Compared with the traditional “wait-and-see” approach, early GPB treatment in selected pre-symptomatic patients may represent a potentially safer management strategy. Such an approach may improve metabolic control, nutritional protein intake, and health-related outcomes in selected patients with later-onset UCD.

## Data Availability

Not applicable.
